# Model for concomitant pulmonary embolism in a deep vein thrombosis patient

**DOI:** 10.1016/j.jvsv.2025.102323

**Published:** 2026-01-08

**Authors:** Saad Ahmad, Rayyan Ali

**Affiliations:** Islamic International Medical College, Riphah International University, Rawalpindi, Pakistan

We read the article “A Predictive Model for Early Detection of Concomitant Pulmonary Embolism in Patients with Deep Vein Thrombosis Immediately Upon Hospital Admission” by Gong et al,^1^ which discusses about early interventions that could be done to determine if a patient has a pulmonary embolism (PE), who is previously diagnosed with deep venous thrombosis (DVT). To address this, it further focuses on reducing the unnecessary computed tomography pulmonary angiography and decreasing the economic burden for patients.

As we further explore the role of detection of concomitant PE in patients with DVT, in cases where a patient presents to the doctor with an already diagnosed DVT, clinicians previously relied on the suspicion of PE from patient's gender, cardiovascular diseases, fracture, age, D-dimer levels, fractures, and iliac vein compression. However, Gong et al created a tool called the nomogram, which demonstrated superior predictive accuracy (about 72.7%) compared with the Wells score, which has a lower accuracy than the nomogram in raising suspicion of PE. The Wells score is based on the clinical suspicion of PE in a patient with DVT; however, machine learning approaches now offer enhanced diagnostic precision over traditional use of the Wells score, increasing accuracy to approximately 80%. Moreover, another advantage of machine learning is that it can be programmed into data systems in the electronic health record, which would be more helpful in early diagnosis, alerting the doctor about the possibility of a PE so that appropriate management and treatment plans can be made. The feasibility of this integration is demonstrated in a study by Zhou et al.^2^

In conclusion, this study provides valuable insights about how machine learning has provided a more valuable tool that enables timely prediction and alerts doctors about the possibility of a PE, which can help doctors to institute an appropriate treatment plan. By enabling earlier detection of key health indicators, this approach has the potential to decrease patient mortality and improve overall outcomes.

## Funding

None.

## Disclosures

None.
